# Impact of High Night Temperature on Yield and Pasting Properties of Flour in Early and Late-Maturing Wheat Genotypes

**DOI:** 10.3390/plants11223096

**Published:** 2022-11-14

**Authors:** Shamima Parveen, Shalini Gaur Rudra, Bhupinder Singh, Anjali Anand

**Affiliations:** 1 Division of Plant Physiology, ICAR-Indian Agricultural Research Institute, New Delhi 110 012, India; 2 Division of Post Harvest Technology, ICAR-Indian Agricultural Research Institute, New Delhi 110 012, India; 3 Division of Environment Science, ICAR-Indian Agricultural Research Institute, New Delhi 110 012, India

**Keywords:** high night temperature, wheat, amylose, amylopectin, rheological properties

## Abstract

The inexorable process of climate change in terms of the rise in minimum (nighttime) temperature delineates its huge impact on crop plants. It can affect the yield and quality of various crops. We investigated the effect of high night temperature (HNT) (+2.3 °C over ambient) from booting to physiological maturity on the yield parameters, grain growth rate (GGR), starch content, composition, and flour rheological properties in early (HI 1544, HI 1563) and late-maturing (HD 2932) wheat genotypes. The change in yield under HNT was highly correlated with grain number per plant (r = 0.740 ***) and hundred-grain weight (r = 0.628 **), although the reduction in grain weight was not significantly different. This was also reflected as an insignificant change in starch content (except in HI 1544). Under HNT, late-sown genotypes (HI 1563 and HD 2932) maintained high GGR compared to the timely sown (HI 1544) genotype during the early period of grain growth (5 to 10 days after anthesis), which declined during the later phase of grain development. The increased rheological properties under HNT can be attributed to a significant reduction in the amylose to amylopectin (AMY/AMP) ratio in early-maturity genotypes (HI 1544 and HI 1563). The AMY/AMP ratio was positively correlated to flour rheological parameters (except setback from peak) under HNT. Our study reports the HNT-induced change in the amylose/amylopectin ratio in early maturing wheat genotypes, which determines the stability of flour starches for specific end-use products.

## 1. Introduction

Wheat contributes 20% of dietary calories to the population worldwide and is consumed as a variety of food products across different regions [1]. The South Asian region contributes to 20.5% of global wheat production [2], albeit with the risk of being targeted by high temperatures during the grain-filling period [3,4]. Gourdji et al. [5] have estimated that by 2030, around 11% of the global area occupied by wheat will be at threat of exposure to mean temperatures >34 °C for at least five days during the reproductive period. Thus, the prediction of heat waves causing an estimated 6–20% yield reduction per degree rise in temperatures in South Asia is a matter of grave concern in the global scenario [6]. India ranks second in the production of wheat and the average yield is reported to be around 30–35 quintals/hectare [7]. The yield penalty of 5.7% experienced this year due to an early onset of high temperatures (>40 °C) during the reproductive phase of the crop was caused due to the shriveling of the grain [8]. A previous analysis of the temperature data has revealed that the increase in daily mean temperatures is reflected as a diurnal asymmetry between the maximum and minimum temperatures [9]. The rate of increase in nighttime temperatures is twice the rate of daytime increase (0.204 °C per decade for nighttime and 0.141 °C per decade for daytime during the period of 1950 to 2004) [10,11]. A high correlation between increased daily mean temperature and reduced starch biosynthesis resulting in shriveled grains has been earlier reported in many studies [12,13,14,15]. Moreover, investigations have found lower grain weight under high night temperatures (HNTs) in wheat [16,17,18,19]. Besides grain weight, the milling quality of grain may be compromised due to changes in the properties and components of the starch [12,14]. Starch consists of glucose polymers, i.e., linear amylose and branched amylopectin, the ratio of which regulates its swelling properties and the ability to form paste upon the uptake of water [20,21].

HNT takes a dual toll on the plants as it causes a decrease in the net rate of photosynthesis accompanied by an increase in respiration [16,18,19,22]. The depletion in the levels of leaf starch during HNT to support high respiration may restrict the carbon supply to the developing grain [19]. Warmer ear temperatures may also impair the carbon balance and disrupt the carbon assimilation in the developing grain [23]. The flour quality (rheological property) of such starch formed under HNT has not been so far studied and gains importance as it affects the different end-use products consumed by customers. Our present study was conducted to evaluate the impact of HNT on the yield and rheological properties of wheat flour in Indian wheat genotypes differing in their maturity durations.

## 2. Results

### 2.1. Temperature Data

**Figure 1:**
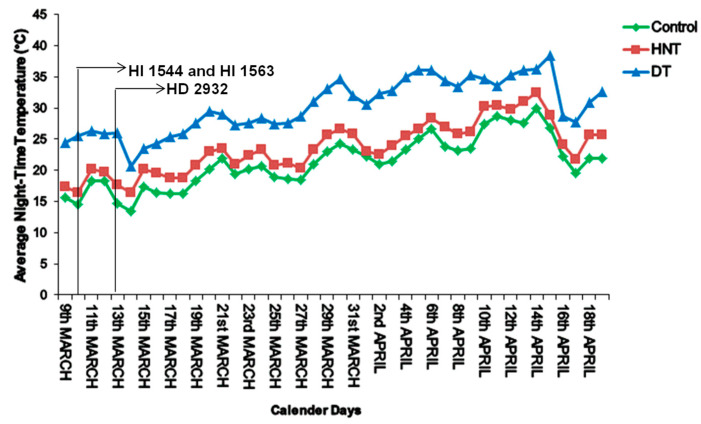
Average nighttime (18:00 to 06:00 h) temperatures in control and HNT chambers during the grain growth period of all the genotypes. DT represents daytime temperature during the same period. The arrows on the *X*-axis represent the time of anthesis.

The mean nighttime temperature between 18:00 and 06:00 h in the control and HNT chambers was 21.3 °C and 23.6 °C, respectively. Thus, a difference of 2.3 °C was maintained from heading to physiological maturity for the wheat genotypes growing under the two environments (Figure 1). The mean daytime temperature during the grain growth period was 30.2 °C for both treatments.

### 2.2. Effect of HNT on Ear Temperature Depression (ΔT Ear)

**Figure 2:**
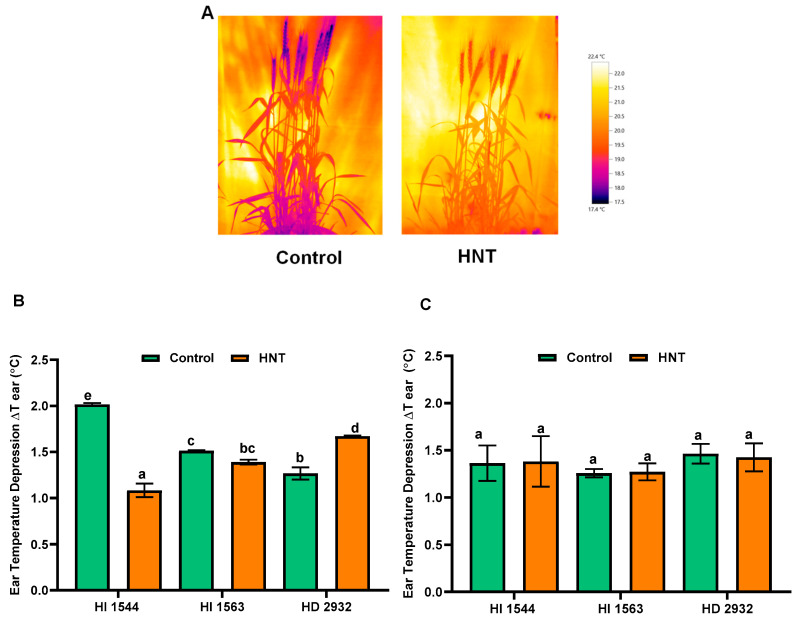
(**A**) Infra-red thermal images of HI 1563 under control and HNT conditions at 10 DAA. (**B**,**C**) Ear temperature depression of wheat genotypes grown under control and HNT conditions at 10 and 20 DAA. Ear temperature depression was defined as the difference between air temperature and ear temperature. Values show mean ± SE. Data were obtained from three replications of each genotype. The separation of means was carried out using Duncan’s post hoc test (*p* < 0.05). Means with different letters are significant.

Genotypic variation was observed for ΔT ear under HNT compared to control at 10 DAA (Figure 2A,B). The early maturing genotypes HI 1544 and HI 1563 showed a reduction (46.3% and 8.1%, respectively) in ΔT ear (increase in ear temperature over ambient) under HNT, though it was significant only for HI 1544. On the other hand, the late-maturing HD 2932 showed a significant increase (32.0%) in ΔT ear (decrease in ear temperature over ambient) under HNT at 10 DAA. The values were not significantly different under the HNT condition across genotypes at 20 DAA (Figure 2C).

### 2.3. Effect of HNT on Gas Exchange Parameters and Stomatal and Non-Stomatal Constraints of Photosynthesis

HNT significantly reduced the net rate of P_n_ in the range of 26.1% to 40.3% and 36.5% to 39.4% in all genotypes compared to their respective controls at 10 and 20 DAA, respectively (Table 1). The nighttime R_n_ rate increased markedly in all genotypes at 10 DAA. However, a significant change in rate was observed at 20 DAA in the early maturing genotypes. The stomatal conductance (G_s_) decreased in the range of 56.8% to 81.6% in all genotypes at 10 DAA under HNT but the values were stable at 20 DAA across treatments and genotypes.

**Figure 3:**
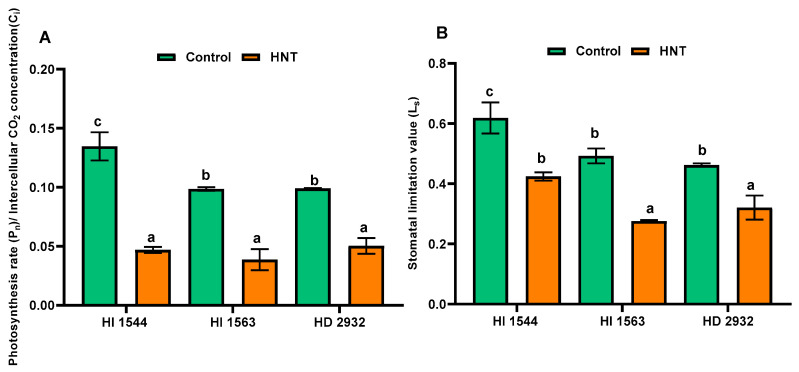
(**A**) Ratio (P_n_/C_i_) of photosynthesis rate (P_n_) to intercellular CO_2_ concentration (C_i_) and (**B**) stomatal limitation value (L_s_) in the flag leaves of wheat genotypes grown under control and HNT conditions at 10 DAA. Values show mean ± SE. Data were obtained from three replications of each genotype. The separation of means was carried out using Duncan’s post hoc test (*p* < 0.05). Means with different letters are significant.

The non-stomatal limitation (P_n_/C_i_ ratio) decreased under HNT at 10 DAA with a major reduction observed for HI 1544 (65.2%) followed by HI 1563 (60.7%) and HD 2932 (49.2%) (Figure 3A). The value of stomatal limitation (L_s_) decreased under HNT compared to control in all genotypes (Figure 3B). The highest reduction was observed in HI 1563 (44.0%) and a similar decline in the range of 30.5–31.4% in the other two genotypes.

**Figure 4:**
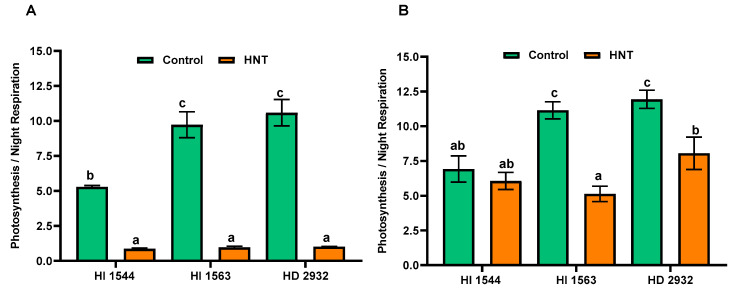
Ratio of photosynthesis to night respiration in the flag leaves of wheat genotypes grown under control and HNT conditions at (**A**) 10 and (**B**) 20 DAA. Values show mean ± SE. Data were obtained from three replications of each genotype. The separation of means was carried out using Duncan’s post hoc test (*p* < 0.05). Means with different letters are significant.

HNT significantly reduced the ratio of P_n_/R_n_ in all genotypes compared to their respective controls, both at 10 and 20 DAA (88.9% and 35.8%, respectively), except in the early maturing genotype HI 1544 at 20 DAA (Figure 4A,B). Amongst the three genotypes, more or less similar ratios (0.87 to 1.01) were observed at 10 DAA under HNT. The highest P_n_/R_n_ was observed in late-sown genotypes under control conditions (HI 1563 and HD 2932) both at 10 and 20 DAA.

### 2.4. Effect of HNT on Grain Growth Rate

**Figure 5:**
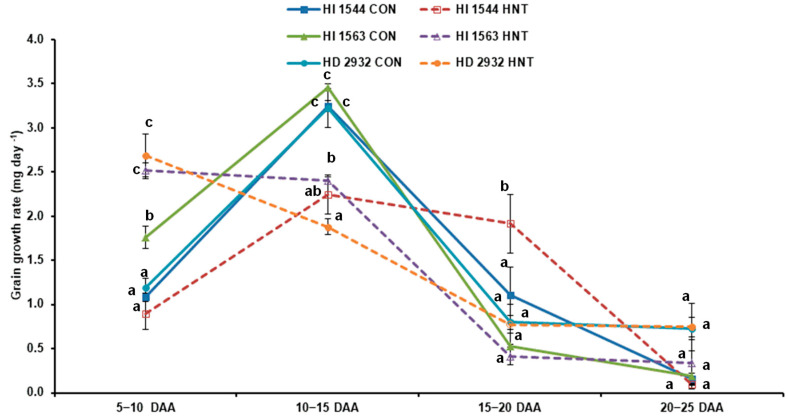
Grain growth rate of wheat genotypes grown under control (CON) and HNT conditions at 5–10, 10–15, 15–20, and 20–25 DAA. Values show mean ± SE. Data were obtained from three replications of each genotype. The separation of means was carried out using Duncan’s post hoc test (*p* < 0.05). Means with different letters are significant.

A significant genotype X environment effect was observed in the early phase of grain development, i.e., 5–10 and 10–15 DAA. At later stages, this effect was diminished as the GGR of all the genotypes (except HI 1544 between 15 and 20 DAA) was not significantly different between the two growing environments. The late-sown genotypes HI 1563 and HD 2932 showed an increased grain growth rate (GGR) of 43.0% and 125.4% under HNT compared to the control at the early grain-filling stage (5–10 DAA). However, all the genotypes manifested higher GGR in ambient conditions than HNT at the later period of accumulation (10–15 DAA), which was also the highest (3.0–3.5 mg day^−1^) during the entire period of grain growth. Between 15 and 20 DAA, a sharp decline in GGR was observed in all the genotypes except HI 1544 under the HNT condition (Figure 5). In HI 1544, the decrease in rate was observed post-20 DAA.

### 2.5. Effect of HNT on Plant Biomass and Yield Components

**Figure 6:**
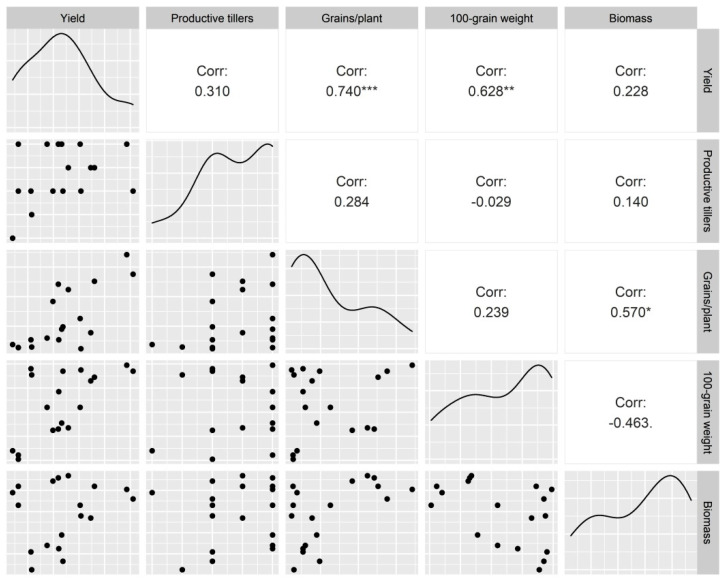
Correlation of growth and yield components at harvest. * Significantly different at *p* < 0.05; ** significantly different at *p* < 0.01; *** significantly different at *p* < 0.001.

Plant biomass reduced significantly under HNT in the late-maturing HD 2932 (8.50%), and the interaction between genotype and environment was significant at *p* < 0.05 (Table 2). Grain yield per plant was reduced by 14.0% in HD 2932 and 23.4% in HI 1544 when plants were subjected to elevated night temperatures after booting. On the contrary, a 15.1% increase in yield under HNT in comparison to control was observed in HI 1563. The heat susceptibility index indicates that the genotypes could be ranked susceptible to HNT in the order HI 1544 > HD 2932 > HI 1563 (Table 3). The number of productive tillers and hundred-grain weight remained unaffected in HNT treatments across genotypes but a significant reduction in grain number per plant was observed in HI 1544 (22.7%) and HD 2932 (24.0%). A genotypic variation was observed for hundred-grain weight, but the response to elevated night temperature was not significant for treatments within each genotype (Table 2). The correlation of yield and its components revealed a significant positive correlation of yield with grain number per plant (r = 0.740 ***) and hundred-grain weight (r = 0.628 **) (Figure 6).

### 2.6. Effect of HNT on Starch Content and Composition

**Figure 7:**
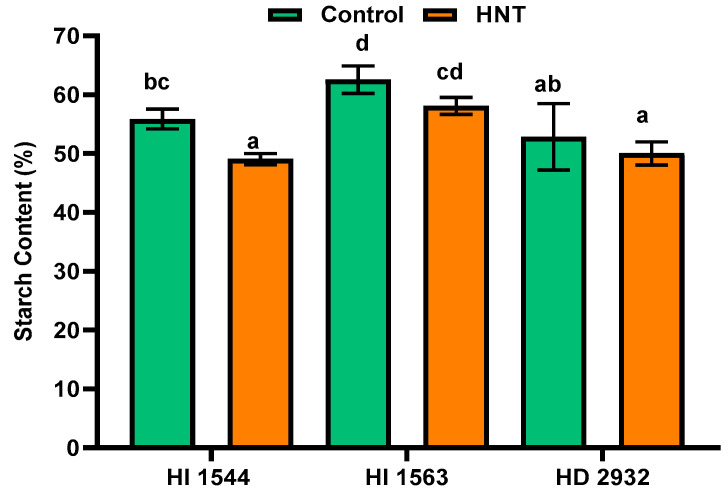
Starch content (%) in the grain of wheat genotypes grown under control and HNT conditions at 20 DAA. Values show mean ± SE. Data were obtained from three replications of each genotype. The separation of means was carried out using Duncan’s post hoc test (*p* < 0.05). Means with different letters are significant.

Starch content decreased significantly in grains under HNT in early maturing genotype HI 1544 (7.42%) compared to the control conditions (Figure 7). A non-significant decline in starch content was observed in late-sown genotypes HI 1563 and HD 2932. HNT also caused a significant reduction and increase in amylose and amylopectin content, respectively, in the early maturing genotypes HI 1544 and HI 1563 (Table 4). However, in late-maturing HD 2932, the amylose and amylopectin content remained stable under HNT compared to the control conditions. Consequently, a significant decline was observed in the amylose to amylopectin ratio (AMY/AMP) in early maturing genotypes HI 1544 (30.13%) and HI 1563 (52.27%) under HNT (Table 4).

### 2.7. Effect of HNT on Flour Quality

**Figure 8:**
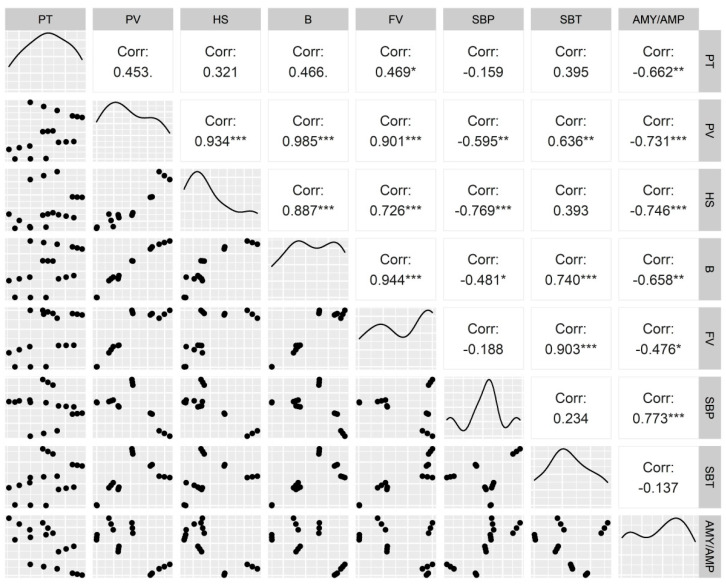
Correlation between the flour rheological properties and amylose to amylopectin ratio of wheat genotypes grown under control and HNT conditions. (PT—pasting temperature; PV—peak viscosity; HS—holding strength; B—breakdown; FV—final viscosity; SBP—setback from peak; SBT—setback from trough; AMY/AMP—amylose to amylopectin ratio). * Significantly different at *p* < 0.05; ** significantly different at *p* < 0.01; *** significantly different at *p* < 0.001.

The rheological properties indicated that HNT significantly affected the flour quality, though genotypic variation was noted (Table 5). A significant increase in PV (peak viscosity), HS (holding strength), B (breakdown), SBT (setback from trough), and FV (final viscosity) were observed under HNT in early maturing genotypes HI 1544 and HI 1563, while late-maturing HD 2932 depicted an opposite trend or remained unaltered. HNT did not significantly affect the PT (pasting temperature) in HI 1544 and HD 2932, though a significant increase was observed in HI 1563. A stable decline in SBP (setback from peak) was observed under HNT in all genotypes compared to their control. The correlation of AMY/AMP with all the rheological parameters indicated a significant negative correlation of AMY/AMP with PT (r = −0.662 **), PV (r = −0.731 ***), HS (r = −0.746 ***), B (r = −0.658 **), and FV (r = −0.476 *) and a positive correlation with SBP (r = 0.773 ***) (Figure 8).

## 3. Discussion

An increase in temperature (daily mean, day or night) at the grain-filling stage is a major deterrent to the stability of yield and quality in wheat [17,18,19,24,25,26]. Early maturity is a favorable strategy in genotypes growing under high temperatures as plants increase their grain growth rate (GGR) to avoid prolonged exposure to high temperatures [6,27]. In our study, a comparison of early and late-maturing genotypes showed that HNT reduced the yield of early maturing, timely sown genotype HI 1544. It demonstrated a reduced GGR from 0 to 15 DAA followed by an increase at a later phase (15–20 days), which, however, could not improve the hundred-seed weight. In contrast, amongst the late-sown genotypes, the negative impact of HNT was observed for the late-maturing HD 2932, whereas HI 1563 (early maturing) showed a significantly higher yield. Both of these genotypes had higher GGR under HNT compared to ambient between 5 and 10 DAA, which may be due to an early onset of starch deposition under high temperatures [28]. This trend discontinued after 10 DAA in HD 2932 but was maintained until 15 days in HI 1563, suggesting its adaptation through an increase in sink activity under HNT. Nevertheless, the differences in GGR could not be realized in terms of the increase in hundred-grain weight in all the genotypes under study, contradicting an earlier report that linked the increase in potential grain weight to grain-filling rate under high temperatures in wheat [29]. The experimental temperatures differed from our study as the effect of both high day and night temperatures (mean day/night temperatures of 25/14 °C vvs 31/20 °C) at anthesis on GGR was evaluated. Our study showed that the change in yield under HNT was highly correlated with grain number per plant (r = 0.740 ***) and hundred-grain weight (r = 0.628 **), although the reduction in grain weight was not significantly different. Similar results have been reported in a study conducted by exposing the plants for a period extending from the third stem node to the 10 DAA stage with a 3.9 °C increase in night temperature over ambient that resulted in a reduction in yield and ear number per unit area [17]. On the contrary, a field experiment with a ~2.4 °C increase in night temperature over ambient from sowing to maturity improved the number of ears per unit area and yield in spring wheat [30]. The varied response of the genotypes to HNT exposure at different stages of growth confirmed the interaction of genotype x environment, which caused a decrease/increase in grain number due to altered gametogenesis, pollen viability, or stigma receptivity [31,32].

The availability of the photosynthate for grain development results from the difference between carbohydrates accumulated during photosynthesis and those respired during respiration [33,34]. It is known that physiological processes, viz., photosynthesis and respiration, are dependent on temperature during crop growth [35,36]. The net rates of photosynthesis and nighttime respiration were severely affected under HNT in all the genotypes at observations taken during the grain-filling stage. Previously, the increased rate of night respiration in wheat, cotton, and rice [18,22,37,38,39,40] have been attributed to higher metabolic requirements due to the increased translocation of photoassimilates to the developing grain, increased sink activity for sugar unloading, and higher maintenance respiration under high temperature [40]. Moreover, photosynthesis was negatively impacted by HNT in wheat and sorghum [16,18,19]. The reduced rate of photosynthesis in these crops occurred due to reduced chlorophyll index, maximum fluorescence yield, and increased thylakoid membrane damage, i.e., non-stomatal limitation as a result of an increase in ROS production under HNT [16,18,30]. In our experiment, the HNT-induced reduction in photosynthesis of all the genotypes during the early stages of grain filling (10 DAA) was due to both stomatal and non-stomatal factors. The stomatal limitation was evidenced by significantly lower values of G_s_ under HNT compared to their respective controls and the non-stomatal limitation was due to lower L_s_ and P_n_/C_i_ ratio. The reduction in the P_n_/C_i_ ratio indicates reduced carboxylation efficiency of Rubisco or a reduced ability for RuBP regeneration [41]. At 20 DAA, the reduction in P_n_ was mainly due to early senescence and non-stomatal limitation, as no significant change was observed in stomatal conductance across genotypes. In previous studies on drought stress, stomatal limitation explained the early-stage inhibition of P_n_, whereas non-stomatal factors were responsible for later stages of grain growth [41,42].

The computation of P_n_/R_n_ ratios showed decreased values under HNT due to a higher increase in respiration rates. However, a reduction in seed weight was not observed in all the genotypes, which suggests that photosynthate was not limiting. Instead, a simultaneous reduction in the number of grains led to the photosynthate being readily available to a lesser number of grains, ultimately helping in the maintenance of seed weight. Borrás et al. [43] analyzed the change in seed dry weight by changing the source (photosynthate availability) during grain growth in wheat, maize, and soybean and observed that sink capacity rather than source availability limited the yield in these crops.

Besides the worldwide concerns about yield reduction under high temperatures, the quality of wheat flour, which depends on the grain protein and rheological/pasting properties of the starch, may also be compromised under increasing temperatures. Our study showed a significantly higher grain protein content in HI 1544 (3.51%) and a non-significant change in HI 1563 (2.44%) and HD 2932 (1.96%) under HNT compared to their respective controls. On the other hand, a significant reduction in the amylose content of early maturing genotypes was observed under HNT. The key enzyme involved in amylose biosynthesis is granule-bound starch synthase I (GBSSI) [44], which was reported to show an abundance of its transcript under HNT [19]. The increased activity of the starch breakdown enzyme, alpha-amylase, has also been implicated in lower starch values under elevated night temperatures [19]. The reduced starch and amylose content of HI 1544 may be an outcome of the reduced GBSS I and/or the increased activity of alpha-amylase at HNT, as the value of ear temperature was higher/ΔT ear was lower compared to other genotypes. However, a non-significant change in ΔT ear and starch content in HI 1563 indicated that alpha-amylase activity may be unaffected under HNT. Furthermore, the exceptional decrease in amylose while maintaining similar ear temperature in this genotype is intriguing and can be unveiled by studying the transcriptional or post-transcriptional regulation of GBSS I in this genotype. Late-maturing genotype HD 2932 showed lower IR temperature of the ear (high ΔT ear) with stable starch and amylose content under HNT. The threshold level of night temperatures above which the activity of the enzymes involved in starch biosynthesis and breakdown are affected needs to be investigated to confirm the relation of T ear, alpha-amylase, GBSS I, and starch metabolism in the developing grain.

The pasting properties of starch determine its end-use quality and are affected by the molecular properties of amylose (chain length, branching ratio, and molecular mass), the ratio of amylose/amylopectin (AMY/AMP), the degree of the polymerization of amylopectin, and the size of starch granules [45,46,47]. They are adversely affected by extreme temperatures and drought during the grain-filling stage [48]. The effect on the functional properties is dependent on the swelling of starch upon the uptake of water (gelatinization), followed by the melting of the crystalline structure and the leaching of amylose molecules (pasting). Following this process, the cooling stage results in the formation of a viscous gel, during which the re-association of amylose and amylopectin into an ordered structure (retrogradation) occurs [49]. The amylose content and, likewise, the AMY/AMP ratio of early maturing wheat genotypes (HI 1544 and HI 1563) reduced significantly under HNT, affecting the various pasting parameters, as captured on the pasting curve. HNT caused an increase in peak viscosity for early maturing genotypes due to the higher proportion of amylopectin leading to more swelling of the starch granules owing to the higher entanglement of branched molecules of amylopectin [50]. The breakdown values in RVA analysis are associated with the disruption of starch during heat application and mechanical shear resulting in amylose leaching out of starch granules. Higher breakdown values in early maturing genotypes under HNT caused s lesser disintegration of starch and lower leaching of amylose from swollen starch granules. The reduced leaching of amylose has a lesser capacity to impede the expansion of starch, resulting in higher swelling, lesser free moisture, and, thus, elevated pasting parameters. Lower setback viscosities in the genotypes under HNT indicated an increased tendency of the AMY present in the hot paste to re-associate during the cooling process [51]. The increase in holding strength under elevated night temperature was also dependent on amylose exudation, granule swelling, and amylose–lipid complex formation [52]. Thus, a significant negative correlation of AMY/AMP was observed with PT (r= −0.662 **), PV (r = −0.731 ***), HS (r = −0.746 ***), B (r = −0.658 **), and FV (r = −0.476 *) and a positive correlation (r = 0.773 **) with SBP, which is in agreement with a previous study [53].

The pasting properties of starch largely determine the biscuit- and cookie-making quality. Most starch pasting parameters such as peak viscosity, breakdown, final viscosity, and setback viscosity are positively linked to spread ratio, sensory scores, specific volume, height, and crumbliness and negatively with hardness and thickness attributes during biscuit-making [54,55,56]. Thus, the increased value of peak viscosity, final viscosity, setback from trough, breakdown, and holding strength in early maturing genotypes (HI 1544 and HI 1563) under HNT may impart favorable attributes to the flour for biscuit-making. To the best of our knowledge, our study is the first report on the reduction in amylose/amylopectin in the wheat flour of early maturing wheat genotypes under HNT, which influences the pasting properties of starch that can affect the processing of wheat flour.

## 4. Materials and Methods

### 4.1. Experimental Site

The present study was conducted in pots kept in HNT chambers at ICAR—Indian Agricultural Research Institute IARI greenhouse facility, New Delhi, India. The dimension of the chambers was 7.3 × 3.7 × 2.1 m (length × breadth × height) and they were fitted with four ceramic heaters and blowers for air circulation. The temperature of the chamber was maintained at ±2–3 °C above the ambient night temperature from 18:00 to 06:00 h.

### 4.2. Plant Materials and Growth Conditions

Three spring wheat genotypes, HI 1544 (timely sown and early maturing), HI 1563 (late-sown and early maturing), and HD 2932 (late-sown and late-maturing), were selected for the study. All the genotypes were sown under very late sowing conditions (last week of December) in the greenhouse facility during 2018–2019. The pots were filled with ten kg of soil mixed with FYM and vermicompost and five seeds were sown per pot. Ten pots were maintained for each genotype. The recommended dose of NPK (120:60:40 kg/ha) was applied as urea, single super phosphate, and muriate of potash, respectively. Nitrogen was applied in two split doses, one-third before sowing and two-thirds at the first node stage. The thinning to three plants per pot was carried out at the third leaf stage. The plants were raised under ambient conditions from the sowing to the booting stage. At the booting stage, five pots per genotype were transferred to control and HNT, respectively, where they were maintained until physiological maturity. HNT treatment was provided from 18:00 to 06:00 h, while the daytime temperatures were similar in the two environments. The temperature was logged every 30 min with a temperature and humidity data logger (Novus Productos Electronicos, Canoas, Brazil). The average daily night temperatures (booting to physiological maturity) logged during the night period from 18:00 to 06:00 h were 21.28 °C (Control) and 23.61 °C (HNT), respectively, and are represented in Figure 1.

The data for yield components and flour quality were obtained at harvest. The data for photosynthesis, night respiration, and ear temperature depression were obtained at 10 and 20 days after anthesis (DAA). The data for starch content and composition were measured at 20 DAA to relate it to the physiological process active during that period.

### 4.3. Ear Temperature Depression

The wheat ears (averaged over five plants per genotype) were imaged using an Infrared Thermographic camera (Testo, Lenzkrich, Germany) at 10 and 20 DAA between 21:00 and 22:00 h (Figure 2A). The camera was placed in the chamber 2 h before the measurements to allow the optics of the camera to reach thermal equilibrium with the chamber temperature. The images thus obtained were analyzed using Testo IRSoft Thermography analysis software (Testo, Lenzkrich, Germany). The ear temperature depression (ΔT ear) was calculated by measuring the difference in temperature between the ear (T ear) and its immediate environment (T ambient) for the correction of any variation existing in the immediate environment referred to as ‘calibration reference temperature’.

Ear temperature depression (ΔT ear) = T (ambient) − T (ear)(1)

### 4.4. Gas Exchange Parameters of the Flag Leaf

The gas exchange parameters such as the net rate of photosynthesis (P_n_), respiration (R_n_), and stomatal conductance (G_s_) were measured on the fully expanded flag leaves from three plants per genotype at 10 and 20 DAA using an infra-red gas analyzer (LI-COR, Lincoln, NE, USA). The photosynthesis and stomatal conductance measurements were taken between 10:00 and 12:00 h and night respiration between 21:00 and 22:00 h. For the measurement of the photosynthesis rate, the light intensity was fixed at 1000 μmol m^−2^ s^−1^ and the flow rate was 500 µmol s^–1^; for respiration, it was adjusted to 300 µmol s^–1^. The data obtained were used to determine the ratio of photosynthesis to night respiration. Moreover, the data on the ratio of photosynthesis to intercellular CO_2_ concentration (C_i_), i.e., (P_n_/C_i_) and stomatal limitation values (L_s_), were computed for 10 DAA since at 20 DAA senescence had set in under the HNT environment. The L_s_ value was calculated as given by Song et al. [42].

L_s_ = 1 − C_i_/C_a_ (2)

where L_s_ = stomatal limitation value, C_i_ = intercellular CO_2_ concentration and C_a_ = ambient CO_2_ concentration.

### 4.5. Grain Growth Rate

The ears of all the genotypes were tagged at anthesis and harvested at 5, 10, 15, 20, and 25 DAA between 08:00 and 10:00 h under both control and HNT conditions. The ears were dried at 40 °C until a constant weight was obtained and the weight of twenty-five grains was measured. The grain growth rate was calculated as given by Natu et al. [57].

Grain growth rate = (W_2_ − W_1_)/(T_2_ − T_1_) (3)

where W_2_ = final grain weight, W_1_ = initial grain weight, and (T_2_ − T_1_) is the duration between two measurements of grain growth.

### 4.6. Plant Biomass and Yield Components under HNT

Data on biomass/plant, number of productive tillers/plant, number of grains/plant, grain yield/plant, and hundred-grain weight were recorded from three pots. Each plant was harvested manually by cutting them at the soil level and dry weights (leaf + stem and ears) constituting biomass were recorded. Leaves and stems were dried at 65 °C until a constant weight was obtained. Ears were dried to constant weight at 40 °C and hand-threshed. The data on grain yield per plant and hundred-grain weight were measured.

The heat susceptibility index (HSI) of the three genotypes was calculated for grain yield at the stressed (HNT) compared to the non-stressed environment (control) by using the formula suggested by Fisher and Maurer [58].

HSI = [l − YD/YP]/D(4)

where YD = mean grain yield of the genotype in a stressed (HNT) environment, YP = mean grain yield of the genotype under a non-stressed (control) environment, D = 1 − [mean yield of all genotypes under stress/mean yield of all genotypes under control]

### 4.7. Starch Content

The starch content was determined in harvested grains by following the protocol of Hodge et al. [59]. Dried grains were ground and 1 g of powdered sample was boiled in 95% ethanol for 10 min. The sample was kept overnight and later washed thrice in 80% ethanol to obtain a residue free from soluble sugars. The residue was again ground and 50 mg of the sample residue was hydrolyzed in 1N HCL in a glycerin bath at (112–115) °C for 30 min. The hydrolyzed sample was filtered using Whatman No. 1 filter paper and the volume was adjusted to 100 mL using double-distilled water. The above filtrate was used for starch estimation using an anthrone reagent and absorbance was measured at 620 nm using a UV-Visible spectrophotometer (Analytik Jena, Jena, Germany).

### 4.8. Starch Composition

For the analysis of starch composition (amylose and amylopectin), triplicate samples of wheat flour were defatted with n-hexane (1:3; *w*/*v*) by stirring the mixture continuously for 60–90 min followed by standing the mixture for 30 min and decantation. The above steps were repeated three times and the defatted flour sample was oven-dried overnight at 30–35 °C. The amylose and amylopectin content was estimated following Ahmed et al. [60]. The defatted flour (100 mg) was placed in the reaction vials and 90% DMSO was added to it. The above contents were mixed vigorously for 20 min using a shaker and heated for 15 min in a water bath at 85 °C. The volume of the above contents was adjusted to 25 mL using double-distilled water and an aliquot of 1 mL was taken and mixed with 5 mL iodine/potassium iodide solution, which was further diluted to 50 mL using double-distilled water. The above mixture was allowed to stand at room temperature for 15 min and the absorbance was measured at two wavelengths, i.e., 620 nm and 540 nm, using a UV-visible spectrophotometer (Analytik Jena, Jena, Germany). The amylose content was calculated from a standard curve prepared using mixtures of pure potato amylose (over the range of 0–100% amylose). Amylopectin content (%) was determined by subtracting the percentage of amylose from the percentage of total starch.

### 4.9. Rheological Properties of Wheat Flour Starch

The rheological properties of the triplicate samples of wheat flour were evaluated using a dynamic rheometer (Anton Paar, Graz, Austria), which measures the pasting profile of flour slurry. Flour (3.5 g) at 14% moisture content was placed in an aluminum can containing 25 mL of distilled water. A programmed heating and cooling cycle was used, where samples were held at 50 °C for 1 min, heated from 50 °C to 95 °C at 6 °C min^−1^, held at 95 °C for 5 min, cooled to 50 °C at 6 °C min^−1^ and held at 50 °C for 2 min. The shearing rate was 960 rpm for the first 10 s followed by 160 rpm until the end of the analysis. The parameters measured were peak viscosity, pasting temperature, holding strength, breakdown, setback from the peak, and setback from trough, which were calculated through Rheoplus software version 3.61 (Anton Paar viscosity and rheology, Graz, Austria).

### 4.10. Statistical Analysis

Statistical analysis was carried out on the data obtained for all parameters by performing a one-way analysis of variance (ANOVA) using SPSS software package version 21.0 for Windows (IBM Corp., Armonk, NY, USA). Significant differences by Duncan’s post hoc test at *p*  ≤ 0.05 were performed with mean values. The Pearson correlations for yield and its components, the amylose to amylopectin ratio, and flour rheological properties under control and HNT were computed using the GGally package in R software (RStudio, Boston, MA, USA).

## 5. Conclusions

The reduced yield in HI 1544 (normally sown and early maturing) is manifested by the reduced grain number per plant under HNT. HNT resulted in lower ∆T ear in HI 1544 along with reduced starch and amylose content. A significant decline in the amylose to amylopectin ratio was observed in both of the early-maturity wheat genotypes, which affected the rheological properties of starch, with favorable implications for biscuit making. However, the maintenance of high amylose content and reduced pasting properties in late-maturity genotype HD 2932 under HNT may affect its end-use quality.

## Figures and Tables

**Table 1:** Photosynthesis, night respiration and stomatal conductance of wheat genotypes grown under control (CON) and high night temperature (HNT) conditions. Values show mean ± SE. Data are obtained from three replications for each genotype. The separation of means was carried out using Duncan’s post hoc test (*p* < 0.05). Means with different letters are significant. ** *p* < 0.01; * *p* < 0.05; ns: not significant (*p* ≥ 0.05).

Genotypes (G)	Treatments (T)	Rate of Photosynthesis (P_n_) (µmole CO_2_ m^−2^ s^−1^)		Rate of Night Respiration (R_n_) (µmole CO_2_ m^−2^ s^−1^)		Stomatal Conductance (G_s_) (mol m^−2^ s^−1^)	
		10 DAA	20 DAA	10 DAA	20 DAA	10 DAA	20 DAA
HI 1544	CON	16.8 ± 0.06 ^b^	13.3 ± 0.37 ^b^	3.2 ± 0.09 ^b^	2.0 ± 0.25 ^c^	0.3 ± 0.01 ^d^	0.1 ± 0.00 ^a^
	HNT	10.0 ± 0.11 ^a^	8.2 ± 0.87 ^a^	11.6 ± 0.48 ^c^	1.4 ± 0.02 ^ab^	0.1 ± 0.00 ^b^	0.1 ± 0.00 ^a^
HI 1563	CON	21.8 ± 1.60 ^c^	14.1 ± 0.08 ^bc^	2.3 ± 0.06 ^a^	1.3 ± 0.06 ^a^	0.3 ± 0.01 ^c^	0.1 ± 0.00 ^a^
	HNT	14.9 ± 1.53 ^b^	8.6 ± 0.42 ^a^	15.3 ± 0.33 ^d^	1.7 ± 0.12 ^bc^	0.1 ± 0.00 ^a^	0.1 ± 0.00 ^a^
HD 2932	CON	22.1 ± 1.77 ^c^	15.7 ± 0.56 ^c^	2.1 ± 0.12 ^a^	1.3 ± 0.03 ^ab^	0.4 ± 0.1 ^e^	0.1 ± 0.00 ^a^
	HNT	16.3 ± 0.23 ^b^	10.0 ± 1.38 ^a^	16.1 ± 0.24 ^e^	1.2 ± 0.03 ^a^	0.1 ± 0.00 ^a^	0.1 ± 0.00 ^a^
ANOVA	G	**	*	**	*	**	ns
	T	**	**	**	ns	**	ns
	G × T	ns	ns	**	**	**	ns

**Table 2:** Yield components and biomass of wheat genotypes grown under CON and HNT conditions. Values show mean ± SE. Data were obtained from three replications for each genotype. The separation of means was carried out using Duncan’s post hoc test (*p* < 0.05). Means with different letters are significant. ** *p* < 0.01; * *p* < 0.05; ns: not significant (*p* ≥ 0.05).

Genotypes (G)	Treatments (T)	Yield per Plant (g)	Number of Productive Tillers per Plant	Grain Number per Plant	100 Grain Weight (g)	Biomass (g/Plant)
HI 1544	CON	7.3 ± 0.18 ^c^	7.0 ± 0.58 ^a^	408.3 ± 8.57 ^b^	3.2 ± 0.01 ^a^	25.2 ± 0.15 ^c^
	HNT	5.6 ± 0.07 ^a^	6.0 ± 1.15 ^a^	315.6 ± 1.70 ^a^	2.9 ± 0.02 ^a^	23.6 ± 0.53 ^c^
HI 1563	CON	6.3 ± 0.21 ^b^	6.3 ± 0.88 ^a^	324.0 ± 4.58 ^a^	3.5 ± 0.10 ^b^	17.8 ± 0.69 ^a^
	HNT	7.3 ± 0.05 ^c^	7.3 ± 0.67 ^a^	340.3 ± 6.74 ^a^	3.4 ± 0.13 ^b^	18.5 ± 0.72 ^a^
HD 2932	CON	9.5 ± 0.47 ^e^	7.0 ± 0.58 ^a^	445.0 ± 13.45 ^c^	3.6 ± 0.03 ^b^	23.9 ± 0.36 ^c^
	HNT	8.2 ± 0.14 ^d^	7.0 ± 0.58 ^a^	338.0 ± 14.73 ^a^	3.5 ± 0.09 ^b^	21.9 ± 0.37 ^b^
ANOVA	G	**	ns	**	**	**
	T	**	ns	**	ns	*
	G × T	**	ns	**	ns	*

**Table 3:** Heat susceptibility indices of wheat genotypes grown under HNT compared to control conditions.

Genotypes	Heat Susceptibility Index
HI 1544	2.61
HI 1563	−1.69
HD 2932	1.56

**Table 4:** Amylose and amylopectin content and their ratios in the grain of wheat genotypes grown under control and HNT conditions at 20 DAA. Values show mean ± SE. Data were obtained from three replications of each genotype. The separation of means was carried out using Duncan’s post hoc test (*p* < 0.05). Means with different letters are significant. ** *p* < 0.01.

Genotypes (G)	Treatments (T)	Amylose Content (%)	Amylopectin Content (%)	Amylose/Amylopectin (AMY/AMP)
HI 1544	CON	26.7 ± 0.46 ^c^	73.3 ± 0.41 ^c^	0.37 ± 0.01 ^c^
	HNT	20.5 ± 0.73 ^b^	80.2 ± 1.43 ^d^	0.26 ± 0.01 ^b^
HI 1563	CON	30.5 ± 0.39 ^d^	69.5 ± 1.21 ^b^	0.44 ± 0.01 ^bc^
	HNT	17.3 ± 0.40 ^a^	82.7 ± 0.40 ^e^	0.21 ± 0.00 ^a^
HD 2932	CON	33.2 ± 0.89 ^e^	66.9 ± 0.05 ^a^	0.50 ± 0.02 ^de^
	HNT	34.3 ± 1.58 ^e^	65.8 ± 1.58 ^a^	0.52 ± 0.02 ^e^
ANOVA	G	**	**	**
	T	**	**	**
	G × T	**	**	**

**Table 5:** Rheological properties of wheat flour in genotypes grown under control (CON) and HNT conditions. Values show mean ± SE. Data were obtained from three replications of each genotype. The separation of means was carried out using Duncan’s post hoc test (*p* < 0.05). Means with different letters are significant. ** *p* < 0.01; ns: not significant (*p* ≥ 0.05).

Genotypes (G)	Treatments (T)	Pasting Temperature (PT) (°C)	Peak Viscosity (PV) (mPa·s)	Holding Strength (HS) (mPa·s)	Breakdown (B) (mPa·s)	Setback from Peak (SBP) (mPa·s)	Setback from Trough (SBT) (mPa·s)	Final Viscosity (FV) (mPa·s)
HI 1544	CON	69 ± 0.1 ^bc^	2140 ± 11.0 ^b^	677 ± 18.5 ^b^	1463 ± 29.8 ^b^	936 ± 8.0 ^c^	2400 ± 21.6 ^b^	3076 ± 3.3 ^b^
	HNT	68 ± 0.2 ^abc^	3495 ± 127.8 ^e^	1294 ± 48.8 ^d^	2260 ± 30.2 ^e^	402 ± 43.4 ^a^	2662 ± 13.0 ^c^	3897 ± 84.5 ^c^
HI 1563	CON	68 ± 0.3 ^ab^	1496 ± 6.1 ^a^	501 ± 7.5 ^a^	994 ± 1.4 ^a^	1016 ± 4.9 ^c^	2011 ± 6.1 ^a^	2512 ± 1.2 ^a^
	HNT	69 ± 0.1 ^c^	3104 ± 22.5 ^d^	967 ± 2.7 ^c^	2136 ± 19.6 ^d^	793 ± 7.0 ^b^	2929 ± 12.6 ^d^	3896 ± 15.5 ^c^
HD 2932	CON	68 ± 0.1 ^abc^	2548 ± 12.2 ^c^	711 ± 15.7 ^b^	1838 ± 3.7 ^c^	1411 ± 44.5 ^d^	3248 ± 47.7 ^e^	3959 ± 32.2 ^c^
	HNT	68 ± 0.2 ^a^	1917 ± 52.3 ^b^	612 ± 77.9 ^ab^	1424 ± 33.8 ^b^	1046 ± 13.0 ^c^	2470 ± 46.9 ^b^	2963 ± 65.73 ^b^
ANOVA	G	ns	**	**	**	**	**	**
	T	**	**	**	**	**	**	**
	G × T	**	**	**	**	**	**	**

## Data Availability

The data sets generated for this study are available on request to the corresponding author.
